# Effects of 24-week treatment with acarbose on glucagon-like peptide 1 in newly diagnosed type 2 diabetic patients: a preliminary report

**DOI:** 10.1186/1475-2840-12-73

**Published:** 2013-05-04

**Authors:** Miao-yan Zheng, Ju-hong Yang, Chun-yan Shan, Hong-tao Zhou, Yan-guang Xu, Ying Wang, Hui-zhu Ren, Bao-cheng Chang, Li-ming Chen

**Affiliations:** 1 Key Laboratory of Hormone and Development (Ministry of Health), Metabolic Disease Hospital & Tianjin Institute of Endocrinology, Tianjin Medical University, Tianjin 300070, China

**Keywords:** Glucagon-like peptide 1, Carotid intima-media thickness, Nitric oxide type 2 diabetes, Acarbose

## Abstract

**Background:**

Treatment with the alpha-glucosidase inhibitor (AGI) acarbose is associated with a significant reduction the risk of cardiovascular events. However, the underlying mechanisms of this effect are unclear. AGIs were recently suggested to participate in stimulating glucagon-like peptide 1 (GLP-1) secretion. We therefore examined the effects of a 24-week treatment of acarbose on endogenous GLP-1, nitric oxide (NO) levels, nitric oxide synthase (NOS) activity, and carotid intima-media thickness (CIMT) in newly diagnosed patients with type 2 diabetes (T2D).

**Methods:**

Blood was drawn from 24 subjects (14 male, 10 female, age: 50.7 ± 7.36 years, BMI: 26.64 ± 3.38 kg/m^2^, GHbA1c: 7.00 ± 0.74%) with drug-naïve T2D at 0 and 120 min following a standard mixed meal for the measurements of active GLP-1, NO and NOS. The CIMT was measured prior to and following 24 weeks of acarbose monotherapy (mean dose: 268 mg daily).

**Results:**

Following 24 weeks of acarbose treatment, both fasting and postprandial plasma GLP-1 levels were increased. In patients with increased postprandial GLP-1 levels, serum NO levels and NOS activities were also significantly increased and were positively related to GLP-1 levels. Although the CIMT was not significantly altered following treatment with acarbose, a decreased CIMT was negatively correlated with increased GLP-1 levels.

**Conclusions:**

Twenty-four weeks of acarbose monotherapy in newly diagnosed patients with T2D is associated with significantly increased levels of both fasting and postprandial GLP-1 as well as significantly increased NO levels and NOS activity for those patients in whom postprandial GLP-1 levels were increased. Therefore, the benefits of acarbose on cardiovascular risk may be related to its stimulation of GLP-1 secretion.

## Background

Type 2 diabetes (T2D) is known to dramatically increase the risk of cardiovascular disease, such as angina pectoris and myocardial infarction, and it is believed that hyperglycemia itself, especially postprandial hyperglycemia, is an independent risk factor for such diseases [1-4]. The previous studies have shown that treatment with acarbose, an alpha-glucosidase inhibitor (AGI), is associated with a significant reduction in cardiovascular events in a population with T2D and impaired glucose tolerance (IGT) [5,6]. However, the underlying mechanisms of this effect are unclear. Recently, new insights into the possible actions of acarbose on cardiovascular risks have been provided by the incretin concept. Following the intake of AGIs, large amounts of undigested carbohydrates reach the lower portion of the small intestine, which is rich in L-cells that produce glucagon-like peptide 1 (GLP-1) and therefore stimulate a long-lasting increased secretion of GLP-1 [7,8]. GLP-1 can elevate levels of nitric oxide (NO), which is the most important endothelium-derived vasodilator and has a potent anti-atherosclerotic effect [9,10] in the coronary effluent from mouse hearts [11]. Moreover, a recent animal study suggests that voglibose (another AGI) can reduce myocardial infarct size through the stimulation of GLP-1 receptors and the activation of the phosphoinositide 3-kinase-Akt-endothelial NOS pathways [12].

To date, the majority of studies have evaluated the short-term effects of acarbose treatment (up to 2 weeks) on GLP-1; the long-term effects of AGIs on NO and NOS in T2D have not been extensively investigated. In the present study, we aimed to explore whether levels of serum GLP-1 and NO and NOS activity increase following chronic (24 weeks) treatment with acarbose monotherapy in newly diagnosed patients with T2D. We also aimed to evaluate the effect of acarbose on carotid atherosclerosis (as defined by carotid intima-media thickness [CIMT]). It is known that CIMT has an important prognostic value with respect to the development of both cardiovascular diseases and of atherosclerotic lesions in the carotid and peripheral arteries [13,14].

## Methods

### Subjects

This was a prospective, observational study of patients with drug-naive T2D who were commencing acarbose as a monotherapy. Newly diagnosed patients with T2D (aged 30–70 years) who met the World Health Organization diagnostic criteria [15] were recruited from the diabetic outpatient clinic in the Metabolic Disease Hospital of Tianjin Medical University between October 2011 and June 2012. The exclusion criteria were glycosylated hemoglobin Alc (HbA1c >9%), a history of congestive heart failure (NYHA Class III or IV), or severe hepatic (serum alanine or aspartate aminotransferase >100 U/L) or renal disease (estimated creatinine clearance <60 ml/min). The patients with current malignant disease, inflammatory bowel disease, those who were pregnant, women who were breast-feeding and patients who had a history of major psychiatric disease were also excluded. Lifestyle intervention was recommended to all of the eligible patients by the diabetic educator. The patients were also excluded if their blood glucose was restored to normal following 1 month of lifestyle intervention. The study protocol was approved by the Tianjin Medical University Ethics Committee Review Board and was conducted using Good Clinical Practice in accordance with the Declaration of Helsinki. All of the participants provided informed consent.

### Study protocol

On the morning of the first visit (V1), following an overnight fasting for 12–14 h, the patients were asked to attend the investigational unit. The anthropometric assessments were performed, including body mass index (BMI) and blood pressure. The blood was drawn at 0 and 120 min following breakfast (the meal was ingested within 10–15 min) for the measurements of plasma active GLP-1, serum insulin NO and NOS activity, and glycated hemoglobin A1c (GHbA1c). Fasting plasma glucose (FPG) and 2 h postprandial plasma glucose (P2PG) levels were also determined. The breakfast was a standard mixed meal that provided approximately 380 kcal, and the energy load was calculated to be 16.0% fat, 9.5% protein and 74.5% carbohydrate. The blood samples were taken and immediately cooled and centrifuged at 4 C. The plasma was stored at -80°C until analysis. The blood samples for the determination of active GLP-1 were collected into tubes that contained heparin anticoagulant and 0.1 mmol/L sitagliptin phosphate (Beijing HuiKang BoYuan Chemical Tech CO., LTD, Beijing, China) to prevent degradation by the dipeptidyl peptidase-4 (DPP-4) enzyme. Next, the CIMT was measured for all of the patients.

Following V1, the patients were commenced on acarbose tablets (Glucobay^®^; Bayer AG, Leverkusen, Germany), beginning at 75 mg daily. The dose was titrated upwards over a period of 4–18 weeks to the dose that produced satisfactory metabolic control or to the maximum dose that was tolerated by the patient. The acarbose tablets were taken with the first bite of the meal, and the maximum given dose was 300 mg daily. The subjects who experienced gastrointestinal side effects during the period of upward titration were instructed to return to the previously tolerated dose for a further 2 weeks and then to attempt to increase the dosage again if the lower dose was tolerated. During this time, all of the patients were instructed to continue with their diet as previously recommended.

Following V1, the participants were asked to again visit the investigational site on the 4th (V2), 8th (V3), 12th (V4), and 18th week (V5) of the study. On these days, the blood glucose levels were determined, lifestyle advice was offered (as in routine clinical practice), compliance with medication was assessed and further dose titration was recommended, as appropriate. All of the subjects attended the visits at 6 and 24 weeks following the initiation of acarbose monotherapy, and the procedures that were described for V1 were repeated. Telephone contact was maintained with all of the subjects between V1 and V6 to monitor their compliance with the medication and to offer advice regarding problems with medication tolerance.

### Laboratory measurements

Both fasting and 2 h postprandial active GLP-1 levels [GLP-1 (7–36) and (7–37)] were determined using commercial enzyme-linked immunoassay (ELISA) kits from Millipore (Billerica, MA, USA). The detection limit of the ELISA was 2 pmol/L, with an intra-assay coefficient of variation (CV) of 7.8% ~ 9.9% and interassay CV of 8.1% ~ 10.8%. The serum NO activity was detected using the nitrate reductase method with the NO Kit (Nanjing Jiancheng BioEngineering Co., Ltd., Nanjing, China). The generation of NO was determined by measuring the release of nitrite (NO_2_^-^ and NO_3_^-^), which is the stable oxidation product of NO. The NOS kit (Nanjing Jiancheng BioEngineering Co., Ltd., Nanjing, China) was used to measure the activity of NOS in the plasma, in which NOS produces NO from L-arginine and oxygen in addition to two iron complexes (the color reagent), which form a colored material. The absorbance is then measured at a wavelength of 540 nm, and the extinction coefficient used to calculate NOS activity. The serum insulin concentration was analyzed using the Roche E170 electrochemiluminescence immunoassay [Elecsys 2012 insulin kit (Ref12017547), Roche Diagnostics GmbH, Indianapolis, IN, USA].

### Carotid ultrasonography measurements

The carotid arteries were assessed using high-resolution B-mode ultrasonography using GE Logiq 7 ultrasound with a 10-MHz high-resolution transducer (General Electric, Wauwatosa, WI, USA). All of the measurements were performed by a single ultrasonographer using the same equipment and were assessed by a single reader who was blinded to the study question, patient, and follow-up time point. A rapid cross-sectional scanning was performed as a first step to pinpoint the location of the possible plaques. The scan was begun from the proximal portion of the common carotid artery (CCA) toward the bifurcation, followed by a scan of the internal and then the external carotid arteries. This process was followed by a longitudinal scanning of the CCA. A segment of the artery (generally where the vessel walls were most clearly discerned throughout the recording) was magnified to identify a distinct lumen-intima and media-adventitia interface. The CIMT was defined as the distance between the leading edge of the lumen-intima interface and the leading edge of the media-adventitia interface. The regions of interest were defined as 1.0 cm distal to the bifurcation, the bifurcation and 1.0 cm proximal to the internal carotid artery in both near and far walls. Next, for each subject, the mean CIMT was reported as the average of 10 measurements (5 measurements from the right and 5 from the left carotid artery).

### Statistical analysis

The data are expressed as the means and the standard deviation or as numbers and percentages. As the levels of serum insulin were markedly skewed, natural logarithm -transformed values were used. The differences in CIMT, anthropometric and laboratory parameters from baseline to the endpoint were compared using a 2-tailed paired t-test. Pearson correlation analysis was used to assess possible relationships between alterations of plasma GLP-1 and NO levels, CIMT and other laboratory data. The analyses were performed using SPSS windows version 17.0, and *p* < 0.05 was considered to be statistically significant.

## Results

A total of 26 patients entered the trial, 24 (92.3%) of whom completed the trial. Two patients dropped out the study (one was not able to tolerate the abdominal distension and anal exhaust and dropped out at V2, the other patient was lost of follow up at V3 for unknown reasons). The mean age of the 24 subjects (14 male, 10 female) was 50.7 ± 7.36 years. The mean dose of acarbose at visit 6 was 268 mg daily (range: 150–300 mg daily). Glycemic control improved in all of the patients following 6 months of acarbose therapy. However, there was no significant change in body weight or BMI (Table 1).

**Table 1:** Changes in glycemic control and body weight with acarbose treatment

**Variable**	**Pre-treatment**	**Post-treatment**	**Mean changes from baseline (95%CI)**	***p*-value**
GHbA1c (%)	7.00 ± 0.74	6.18 ± 0.51	-0.82(-1.23, -0.41)	0.000
FPG (mmol/L)	8.31 ± 0.93	7.20 ± 0.85	-1.11(-1.56, -0.66)	0.000
P2PG (mmol/L)	14.34 ± 2.65	10.10 ± 1.12	-4.25(-5.56, -2.94)	0.000
Weight (kg)	74.71 ± 12.30	74.96 ± 11.70	-0.25(-0.78, 0.28)	0.341
BMI (kg/m^2^)	26.64 ± 3.38	26.64 ± 0.38	-0.10(-0.29, 0.09)	0.279

Data are expressed as mean ± S.D; HbA1c: glycated haemoglobin A1c; FPG: fasting plasma glucose; P2PG: 2 hour postprandial plasma glucose; BMI: body mass index.

### Plasma GLP-1 and serum insulin levels

#### Plasma GLP-1

**Figure 1:**
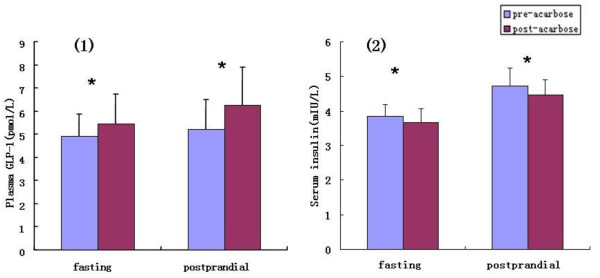
**Mean values for plasma GLP-1 and serum insulin pre- and post-acarbose treatment. **The values of serum insulin was natural logarithm-transformed; *p < 0.05 for the difference between pre- and post-acarbose treatments.

Following 24 weeks of acarbose monotherapy, the fasting plasma GLP-1 levels increased from 4.92 ± 0.94 pmol/L (pre-treatment) to 5.46 ± 1.28 pmol/L (post-treatment) (*P*<0.05). The 2 h postprandial GLP-1 levels increased from 5.23 ± 1.26 pmol/L (pre-treatment) to 6.26 ± 1.64 pmol/L (post-treatment, *P*<0.05, Figure 1(1)).

#### Serum insulin

The mean 2 h postprandial serum insulin (natural logarithm-transformed) levels were significantly reduced following acarbose treatment (post-treatment: 4.48 ± 0.42 mIU/L vs. pre-treatment 4.72 ± 0.53 mIU/L, *p* = 0.041). The mean fasting insulin (natural logarithm-transformed) levels were marginally reduced following the 24-week treatment period, but this difference was not statistically significant (post-treatment: 3.68 ± 0.40 mIU/L vs. pre-treatment: 3.85 ± 0.35 mIU/L, P = 0.064, Figure 1(2)).

#### Serum NO levels and NOS activity

**Figure 2:**
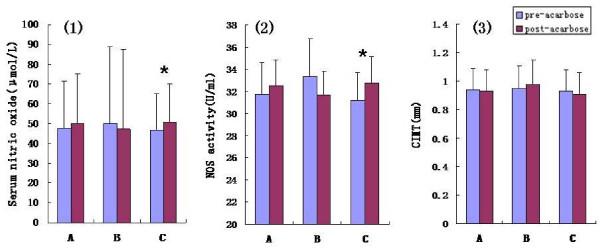
**Difference of serum nitric oxide, NOS activity and CIMT between pre- and post-acarbose treatment. **NOS: nitric oxide synthase; CIMT: carotid intima-media thickness; A: all of the subjects; B: the subjects with decreased postprandial GLP-1 following acarbose treatment; C: the subjects with increased postprandial GLP-1 following acarbose treatment; *p < 0.05 for the difference between the pre- and post-acarbose treatment.

Both the serum NO levels and NOS activity were marginally increased following the 24-week acarbose treatment; however, these changes were not statistically significant (post-treatment NO 49.90 ± 25.07 μmol/L, NOS activity 32.49 ± 2.34 U/ml vs. pre- treatment NO 47.48 ± 24.18 μmol/L, NOS activity 31.75 ± 2.83 U/ml, P>0.05, Figure 2(1, 2) A). We further divided the patients into the following subgroups according to the observed changes in fasting or 2 h postprandial plasma GLP-1 levels: fasting GLP-1 increased group (n = 17) vs. decreased group (n = 7), and postprandial GLP-1 increased group (n = 18) vs. decreased group (n = 6). We observed that both the serum NO levels and NOS activities were significantly increased following acarbose treatment for those in the postprandial GLP-1 increased subgroup (post-acarbose NO 50.78 ± 19.28 μmol/L, NOS activity 32.76 ± 2.40 U/ml vs. pre-acarbose NO 46.61 ± 18.49 μmol/L, NOS activity 31.21 ± 2.50 U/ml, P<0.05, Figure 2(1, 2)C). No such significant change was observed for those in the postprandial GLP-1 decreased subgroup (P>0.05, Figure 2(1, 2)B). There were no significant changes for the fasting GLP-1 increased or decreased subgroups (P>0.05, data not shown).

#### CIMT

The mean CIMT was not significantly different following 24 weeks of acarbose monotherapy (*P*>0.05, Figure 2 (3)A). Moreover, the pre- and post-acarbose treatment CIMTs were not significantly different for either the fasting GLP-1decreased/increased subgroups (data not shown) or for the postprandial GLP-1 decreased/increased subgroups (*P*>0.05, Figure 2 (3)B, C).

#### The correlation between the changes in GLP-1, NO, CIMT and other laboratory data with acarbose treatment

Increased values of fasting GLP-1 were negatively correlated with a change in GHbA1c levels (*R* value: -0.491; P<0.05). Moreover, increased 2 h postprandial GLP-1 levels were negatively correlated with the changes of GHbA1c, FPG and the mean CIMT (*R* values: -0.636, -0.481 and -0.628, respectively; P<0.05) and positively correlated with the changes of NO and NOS (*R* values: 0.452 and 0.561, respectively; P<0.05). The changes in the mean CIMT were negatively correlated with changes in NO levels (*R* value: -0.577; P<0.05) but not with changes in NOS activity (*R* value: -0.314; P = 0.135). Furthermore, the changes in NO levels and NOS activity were positively correlated (*R* value: 0.449; P<0.05) (Table 2).

**Table 2:** Correlations between changes in GLP-1, NO, CIMT and other laboratory data following acarbose treatment

	**Δfasting GLP-1**		**Δ2 h postprandial GLP-1**		**ΔNO**		**ΔNOS**	
	***R *value**	***P *value**	***R *value**	***P *value**	***R *value**	***P *value**	***R *value**	***P *value**
△Weight	-0.143	0.506	0.014	0.950	0.024	0.911	0.121	0.574
△BMI	-0.128	0.550	0.027	0.899	0.038	0.861	0.138	0.519
△GHbA1c	-0.491	0.015	-0.636	0.001	-0.143	0.504	-0.149	0.488
△FPG	-0.185	0.388	-0.481	0.017	0.038	0.859	-0.108	0.617
△P2PG	-0.181	0.398	-0.110	0.608	0.241	0.256	0.007	0.973
△ln Insulin (0 h)	-0.176	0.411	0.145	0.498	0.285	0.177	0.363	0.081
△ln Insulin (2 h)	-0.035	0.871	0.084	0.696	0.135	0.528	0.079	0.713
△mean CIMT(mm)	-0.384	0.064	-0.628	0.001	-0.577	0.003	-0.314	0.135
△NO	0.245	0.249	0.452	0.026	-	-	0.449	0.028
△NOS	0.268	0.205	0.561	0.004	0.449	0.028	-	-

Δ: The change of values following acarbose treatment; ln: natural logarithm -transformed values; BMI: body mass index; HbA1c: glycated haemoglobin A1c; FPG: fasting plasma glucose; P2PG: 2 hour postprandial plasma glucose; CIMT: carotid intima-media thickness; NO: nitric oxide; NOS: nitric oxide synthase.

## Discussion

Our study indicates that chronic acarbose therapy is associated with a significant increase in both fasting and postprandial active GLP-1 levels in newly diagnosed patients with T2D. What’s more, our study provides evidence that postprandial GLP-1 increment after acarbose treatment is closely correlated with increment of NO levels and NOS activity and also with reduction of the mean CIMT. This is, to the best of our knowledge, the first study to investigate the long term effect of AGIs on GLP-1, NO secretion, NOS activity and its association with improved atherosclerosis in newly diagnosed patients with T2D.

### Effects of acarbose on plasma GLP-1 and insulin level

We demonstrated that, in newly diagnosed patients with T2D, chronic acarbose therapy is associated with a significant increase in both fasting and postprandial active GLP-1 concentrations in addition to good glycemic control, as expected. These results are consistent with other studies that have reported increased postprandial GLP-1 levels following administration of either (i) following a single dose of acarbose or following 2 weeks of acarbose treatment in both normal subjects and those with T2D [7,8,16,17], or (ii) acarbose in combination with alogliptin (a DPP-4 inhibitor) [18]. This effect is most likely due to a reduction in carbohydrate absorption in the proximal portion of the small bowel, increasing the load of these nutrients in the distal intestine, where GLP-1 secretion is greater. Unlike previous studies, we demonstrated this effect following long-term (24 weeks) therapy, highlighting yet another important aspect of the glucose-lowering properties of acarbose among new diagnosed patients with T2D. In the present study, we first report that fasting active GLP-1 levels are significantly elevated following 24 weeks of acarbose treatment. As previously reported, the functional integrity of GLP-1-secreting cells is seriously impaired even in the context of mild diabetes, and GLP-1 is deficient in patients with T2D [19,20]. Our data indicate that increased fasting GLP-1 levels are negatively correlated with changes of GHbA1c and that increased 2 h postprandial GLP-1 levels are negatively correlated with the observed changes in both GHbA1c and FPG (Table 2). These results suggest that glycemic control following a 24-week treatment with acarbose may contribute in part to the restoration of the GLP-1 secretion defect in newly diagnosed patients with T2D. Previous studies have focused on the effect of GLP-1 following either a single dose of acarbose or a brief treatment period (i.e., not more than 2 weeks), and only changes in postprandial GLP-1 levels have been reported [6,7,16,17]. The reason that previous studies did not conclusively demonstrate the relationship between fasting GLP-1 and acarbose treatment may be due to the short treatment periods that were tested.

In theory, GLP-1 can stimulate insulin secretion and inhibit the generation of glucagon. However, our data indicate the level of postprandial insulin is significantly reduced (*P*<0.05) following acarbose treatment and that fasting insulin levels are marginally reduced (*p* = 0.064), even in the patients with increased GLP-1 levels. Furthermore, the increased levels of GLP-1 were not correlated with the change in insulin levels (*p* > 0.05). It is known that several factors regulate insulin secretion and that glucose is the most important nutrient secretagogue. The delayed digestion of carbohydrates and cleavage of oligosaccharides by AGIs results in undigested carbohydrates reaching the lower portions of the small intestine and stimulating GLP-1 secretion. This intestinal hormone delays the emptying of the stomach, reduces glucagon secretion, and regulates insulin secretion, which in fact is dependent on blood glucose concentrations. This effect may in part explain why long-term treatment with acarbose results in a reduction in not only postprandial but also fasting blood glucose concentrations [7,21]. Thus, reduced blood glucose concentrations following acarbose treatment may result in (i) markedly lower stimulation of insulin synthesis and insulin secretion and (ii) decreased insulin resistance-induced hyperinsulinemia [22].

### Effects of acarbose on NO, NOS activity and CIMT

As an endothelium-derived vasodilator, NO has been demonstrated to play a crucial role in the development of vascular complications via the regulation of blood flow and various antiatherosclerotic actions [9,10]. CIMT is a widely accepted indicator of subclinical atherosclerosis burden, with higher values being associated with an adverse cardiovascular prognosis. Consequently, CIMT has also been proposed to be a surrogate end point for therapeutic interventions that aim to lower the atherosclerotic burden [23,24]. In this study, we also demonstrated that both serum NO levels and NOS activity are significantly higher following acarbose treatment in the subjects whose postprandial GLP-1 was increased (P<0.05). Moreover, the increase in 2 h postprandial GLP-1 levels was (i) positively correlated with changes in NO levels and NOS activity (R values: 0.452 and 0.561, respectively; P<0.05) and (ii) negatively correlated with the change in the mean CIMT (R value -0.669; P<0.05). It has been reported that GLP-1 participates in the upregulation of the activity and protein expression of NOS in human umbilical vein endothelial cells [25] and improves endothelial dysfunction in patients with T2D and coronary heart disease [26]. What’s more, serum levels of NO metabolites may be a simple, safe, convenient and reliable method for the evaluation of visceral fat accumulation in clinical diagnostic screening [27]. Several clinical studies have shown GLP-1 analogue liraglutide, alone or in combination with insulin, has produced meaningful long-term weight loss, diminished abdominal obesity and significantly improved eating behavior in patients with T2D compared with insulin treatment alone [28,29], which might be beneficial to improving endothelial dysfunction.

In a recent animal study, it has reported that orally administered voglibose, another AGI, protects the myocardium against ischemia–reperfusion injury through the stimulation of GLP-1 receptors, the activation of PI3K-Akt endothelial NOS pathways, and the opening of mitochondrial KATP channels in rabbits after 30 minutes of coronary occlusion and 48 hours of reperfusion [12]. In the present study, the observed changes in CIMT were not significantly different pre- and post-acarbose treatment. In fact, several studies have demonstrated that AGIs treatment is associated with a significant reduction the risk of cardiovascular events in populations with IGT and T2D [5,6,30]. A recent randomized comparable study has shown that AGI, miglitol, can reduce visceral fat accumulation and cardiovascular risk factors in subjects with metabolic syndrome [31]. Combination of miglitol and DPP-4 inhibitor sitagliptin can reduce postprandial glucose fluctuation and stabilize blood glucose levels [32], which may benefit to delaying atherosclerosis [3]. In a subgroup study of the STOPNIDDM trial, for which the progression of CIMT was the primary objective, the annual progression of CIMT in the placebo group was 0.014, whereas this value was 0.007 mm (p < 0.01) in the acarbose group after 3.3 years of follow-up [33]. While we did not observe that CIMT was significantly decreased with elevated GLP-1 or NO levels, although the changes of CIMT were negatively correlated with changes of fasting, postprandial GLP-1 and NO in the present study (*R* values: -0.436, -0.669 and -0.573, respectively; P<0.05). One possible reason for this result may be due to the brief period of the study. Twenty-four weeks is perhaps too short a period over which to observe changes in CIMT.

### Study limitations

The present study has several limitations. First, it is a non-randomized controlled study. Having a placebo arm may have strengthened our study further. What’s more, only 24 patients finished in the study and the sample size is small, which might be a reason that we didn’t observe significant change of NO level and NOS activity although postprandial GLP-1 increment is closely correlated with increment of NO levels and NOS activity after 24-week acarbose treatment. So, further large and randomized controlled study is needed.

## Conclusions

24 weeks of acarbose treatment in newly diagnosed patients with T2D is associated with increased levels of both fasting and postprandial GLP-1. Moreover, increased GLP-1 levels increase NO secretion and NOS activity, potentially explaining the beneficial role of acarbose in preventing cardiovascular disease in these patients.

## Abbreviations

AGI: Alpha-glucosidase inhibitor; BMI: Body mass index; CCA: Common carotid artery; CIMT: Carotid intima-media thickness; CV: Coefficient of variation; DPP-4: Dipeptidyl peptidase-4; ELISA: Enzyme-linked immunoassay; FPG: Fasting plasma glucose; GHbA1c: Glycated hemoglobin A1c; GLP-1: Glucagon-like peptide 1; IGT: Impaired glucose tolerance; NIDDM: Noninsulin-Dependent Diabetes Mellitus; NO: Nitric oxide; NOS: Nitric oxide synthase; P2PG: 2 h postprandial plasma glucose; T2D: Type 2 diabetes.

## Competing interests

The authors declare that they have no conflicts of interest.

## Authors’ contributions

Zheng MY and Chang BC acquired and analyzed data, and wrote the manuscript. Chen LM conceived of the study, analyzed data and reviewed the manuscript. Zhou HT carried out Carotid ultrasonography measurements. Shan CY, XU YG, Wang Y and Ren HZ acquired and researched data. All authors read and approved the final manuscript.
